# Insights into molecular properties of the human monocarboxylate transporter 8 by combining functional with structural information

**DOI:** 10.1186/1756-6614-4-S1-S4

**Published:** 2011-08-03

**Authors:** Gunnar Kleinau, Ulrich Schweizer, Anita Kinne, Josef Köhrle, Annette Grüters, Heiko Krude, Heike Biebermann

**Affiliations:** 1 Institut für Experimentelle Pädiatrische Endokrinologie, Charité-Universitätsmedizin Berlin, Germany; 2 Institut für Experimentelle Endokrinologie, Charité-Universitätsmedizin Berlin, Germany; 3 Leibniz-Institut für Molekulare Pharmakologie, Berlin, Germany

## Abstract

**Background:**

The monocarboxylate transporter 8 (MCT8) is a member of the major facilitator superfamily (MFS) and transports specificly iodothyronines. MCT8 mutations are the underlying cause of a syndrome of severe X-linked psychomotor retardation known as the Allan-Herndon-Dudley syndrome. This syndrome is characterized by abnormally high T3, low/normal T4 serum levels and slightly elevated serum TSH. To date, more than 25 pathogenic mutations in *hMCT8* are known and they are valuable indicators of important regions for structural and functional MCT8 properties.

**Methods:**

We designed a structural human MCT8 model and studied reported pathogenic missense mutations with focus on the estimation of those amino acid positions which are probably sensitive for substrate transport. Furthermore, assuming similarities between determinants of T3 binding observed in the published crystal structure of the thyroid hormone receptor beta occupied by its ligand T3 and the structural MCT8 model, we explore potential T3 binding sites in the MCT8 substrate channel cavity.

**Results:**

We found that all known pathogenic missense mutations are located exclusively in the transmembrane helices and to a high degree at conserved residues among the MCT family. Furthermore, mutations either of or to prolines/glycines are located mainly at helices 9-12 and are expected to cause steric clashes or structural misfolding. In contrast, several other mutations are close to the potential substrate channel and affected amino acids are likely involved in the switching mechanism between different transporter conformations. Finally, three potential substrate binding sites are predicted for MCT8.

**Conclusions:**

Naturally occurring mutations of MCT8 provide molecular insights into protein regions important for protein folding, substrate binding and the switching mechanism during substrate transport. Future studies guided by this information should help to clarify structure-function relationships at MCT8 which may bear broader relevance for other members of the MCT family. This includes decoding of the complete set of transport-sensitive residue positions and description of structural re-arrangements during transport.

## Introduction

The Allan-Herndon-Dudley syndrome is an X-linked mental retardation first described in 1944 [1]. Neonates are lacking head-moving control and they are characterized by feeding problems. Severe psychomotor retardation becomes more obvious with increasing age and some patients never attain speech or the ability to walk. An abnormal thyroid hormone constellation was found as a characteristic signature for this syndrome and guided the identification of mutations in MCT8 in affected patients [2,3]. This constellation is a combination of high T3 levels with low/normal T4 and slightly increased TSH in human serum. To date, more than 25 pathogenic mutations have been identified including missense, nonsense, deletions, insertions, and splice site mutations [4].

MCT8 is a member of the major facilitator superfamily (MFS) of membrane transporters and is structurally characterized by twelve transmembrane helices connected by intra- and extracellular loops. The N- and C-termini are both located intracellularly [5]. MCT8 was described as a specific iodothyronine transporter [6] and known substrates for human MCT8 are thyroxine (3,3’,5,5’-tetraiodo-L-thyronine, T4), T3 (3,3’,5-triiodo-L-thyronine), rT3 (3,3`,5’-triiodo-L-thyronine), and 3,3’-T2 (3,3’-diiodo-L-thyronine) [6,7].

It is assumed that substrate binding by MFS transporters triggers the transition between an “outside-open” and “inside-open” conformation, the so-called “rocker-switch” model [8,9]. This structural movement must be linked with specific intramolecular events like re-arrangement of amino acid interactions and also with substrate trans-localization. Despite the need of more supporting and detailed information for this structural-functional “rocker-switch” model, specific substrate-protein interactions need to be considered as to be causally related to different protein conformations. Of note, this model should be also applicable for the reverse way of substrate transport (i.e. efflux cycle) from the cytosolic to the extracellular site. So far, details and principles of the process are only partially known.

Naturally occurring missense mutations point to functionally and structurally important residues within the MCT8 protein. Mutations may affect protein folding and stability or directly modify the mechanism of iodothyronine transport [10]. We here explored the spatial localization, molecular environment and the potential function of their wild-type amino acids at a three-dimensional “inside-open” MCT8 model, the only structural conformation where templates for homology models are available. We will also discuss the results of previous studies to evaluate the MCT8 homology model and to extent generally our knowledge regarding molecular mechanisms at MCTs.

## Material and methods

### Homology modelling of MCT8

The crystal structure of the Glycerol-3-phosphate transporter (GlpT) in an “inside-open” conformation was used as a structural template for the human MCT8 (hMCT8) homology model (PDB code 1PW4 [8]). Despite low sequence identity between this template and other members of MFS like MCT8, models based on this structural template are supported by experimental data concerning GLUT1 [11], MCT1 [12,13], and OATP1C1 [14]. The potential dimensions of MCT8 transmembrane helices (TMHs) were defined based on helices in the template crystal structure. For consistency we confirmed these dimensions with other available MFS crystal structures (PDB codes 1PV6 and 1PV7 [15]). For further structural information of MCTs see also the internet platform “Membrane transporter systems” at http://www.membranetransport.org.

Gaps of missing residues in loop regions of the template structure were closed either manually or by the ‘Loop Search’ tool implemented in Sybyl 7.35 (Tripos Inc., St. Louis, MO, USA). Side chains and loops of the homology model were subjected to conjugate gradient minimization (until converging at a termination gradient of 0.05 kcal/(mol*Å)). The side chain orientations were refined during dynamic simulation (8 ns) by fixing all backbone H-bonds of TMHs. This step of model generation is extended compared to the recently published MCT8 model [7] (5 ns) to get more precise potential MCT8 side chain orientations. Finally, the model was minimized without any constraints.

**Figure 1:**
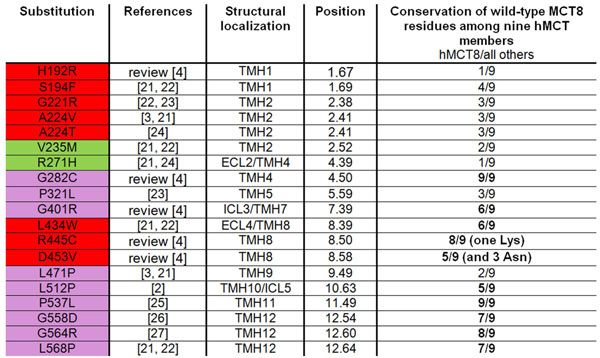
**Naturally occurring missense substitutions in human MCT8.** In this table naturally occurring pathogenic amino acid substitutions known for MCT8 are summarized. Specific background-colours indicate structural/functional and biophysical specificities: violet – mutations at or to proline/glycines, respectively; red – side chain substitutions in the vicinity of the presumed substrate channel (figures 2 and 3); green – amino acids pointing towards the membrane. We also provide a summary on conservation of respective amino acids within the MCT family based on the alignment in figure 4 (bold are positions where more than half of the MCTs have an identical amino acid). The unified position numbering suggested in a recent study [7] is provided (for description see section Material and Methods).

**Figure 2:**
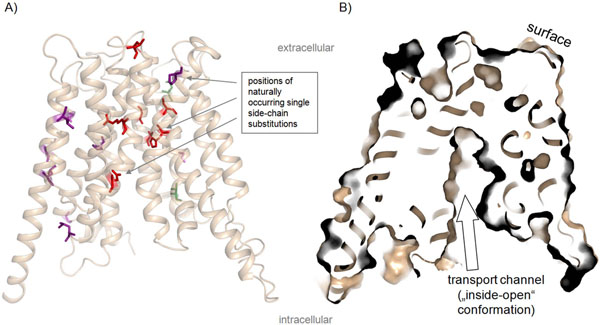
**Homology model of human MCT8 with highlighted naturally occurring side chain substitutions. A)** We highlighted wild-type amino acids of known naturally occurring mutations on the homology model of monocarboxylate transporter 8 in a conformation open to the cytosol (see figure 1). Colours were assigned according to the colour scheme in figure 1. From this three-dimensional mapping it becomes clear that none of these mutations are localized in the extra- or intracellular loops. **B)** The clipped surface-presentation of the MCT8 protein reveals the presumed substrate channel from the intracellular side.

**Figure 3:**
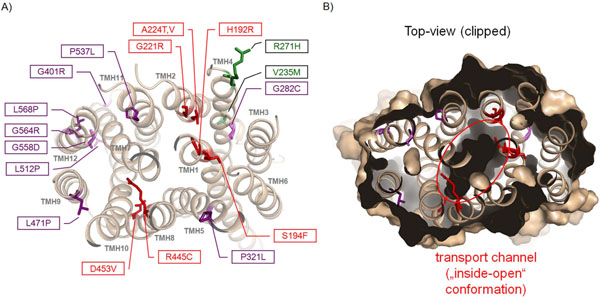
**Homology model of the human MCT8 with mapped details of pathogenic mutations and potential localization of the transport channel switching region. A)** The top-view on the MCT8 homology model with annotated pathogenic side chain substitutions reveals a detailed insight into the distribution of known mutations. The colour code given to the side chains (sticks) is in accordance with described colour scheme in figure 1. **B)** This clipped surface top-view visualizes the spatial relation between the wild-type amino acids of mutant positions and the “inside-open” transport channel conformation. Amino acids that are localized at the transition between the open and closed channel part are coloured red (red circle). These residues might be involved in the switching process between the “inside-open” and “outside-open” conformations.

We have mapped naturally occurring single side chain substitutions (figure 1) on our MCT8 model (figures 2 and 3). Pathogenic deletions or insertions (reviewed in [4]) are not considered here, since they lead to incomplete proteins or they modify the particular helix-conformation and arrangements relative to each other. Therefore, such mutations are revoking a consistent structural prediction.

**Figure 4:**
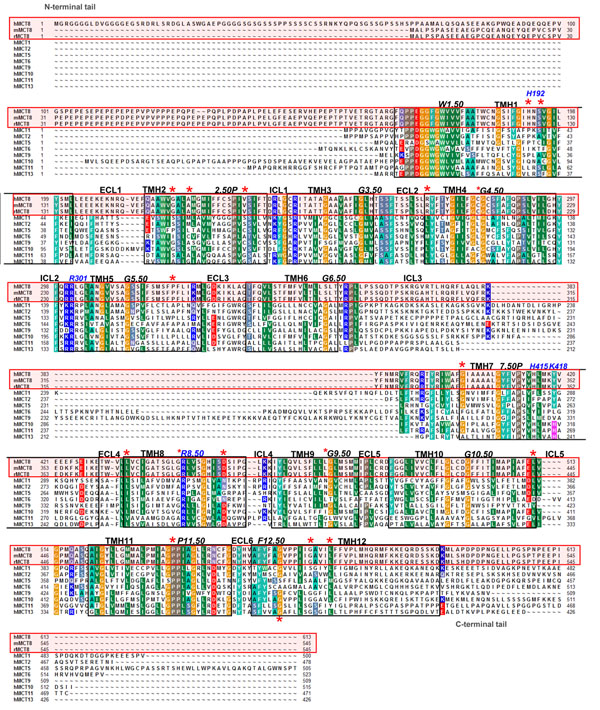
**Alignment of the human MCTs with highlighted positions of pathogenic mutations reported for MCT8**. This alignment compares the human MCT sequences and in addition MCT8 of rat and mice (MCT8 are red boxed). Conserved residues are marked by different colours according to their biophysical properties (blue - positively charged, red - negatively charged, green/orange - hydrophobic, gray - hydrophilic, black – proline, cyan - aromatic). Black boxed are the potential dimensions of the transmembrane helices (TMHs) based on the X-ray structure of the *E.coli* Glycerol-3-phosphate transporter, another member of the major facilitator superfamily which was used as a structural template to build the MCT8 homology model. Red stars above the hMCT8 sequence indicating positions where naturally occurring mutations for the *hMCT8* are reported. Few of them are at positions with a high degree of conservation like R445 (R8.50). In addition, the positively charged residues and histidines hypothesized in this study as to be potentially involved in substrate transport (figure 7) are annotated above the sequences in blue. For each helix the most highly conserved residue is numbered according to a unifying numbering system (see Material and Methods).

**Figure 5:**
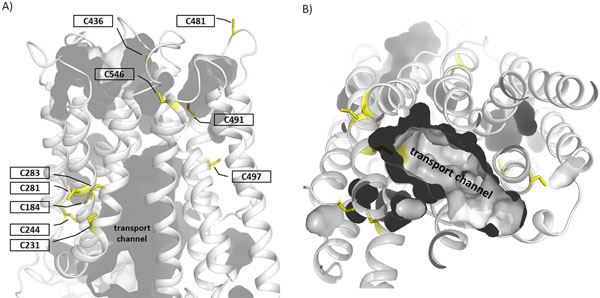
**Distribution of cysteines in the human MCT8 homology model**. To evaluate our MCT8 model on consistency we examined already published functional data. Alanine mutants of all ten cysteines in the hMCT8 did not alter MCT8 functions such as substrate transport [16]. **A)** With the exception of C283 at the cytosolic channel region our “inside-open” MCT8 model suggests that none of the MCT8 cysteine side chains directly participates in the substrate channel. In conclusion the experimental data and the implications from the MCT8 model are in agreement with each other. **B)** The cytosolic view with clipped intracellular loops shows that most of the cysteines are localized at distance from the substrate channel. (Cavities in the model are highlighted by inner surfaces.)

**Figure 6:**
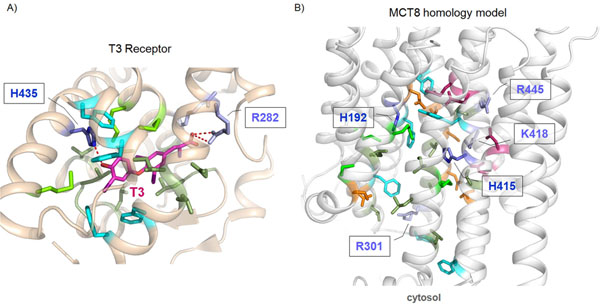
**Details of the T3 receptor beta complex in comparison with determinants of the MCT8 transport channel. A)** The crystal structure of the T3 receptor beta ligand binding domain (PDB code 3GWS [20] backbone-ribbon) reveals detailed insights into the binding mode of this hormone. Surrounded by hydrophobic (green) and aromatic (cyan) amino acids, T3 (magenta) binding is characterized by H-bonds to an arginine and a histidine. According to the assumption of analogy for T3 binding (T3 receptor, A) to different proteins, such specific complementary residues might be generally mandatory for hormone binding/transport. **B)** In the MCT8 homology model two histidines (H192, H415) and three positively charged residues (R445, K418, R301) are predicted to point inside the putative substrate transport channel (sticks with labels). Further amino acids in the transport channel are also highlighted by sticks: orange – hydrophilic residues without charges, magenta – negatively charged residues.

**Figure 7:**
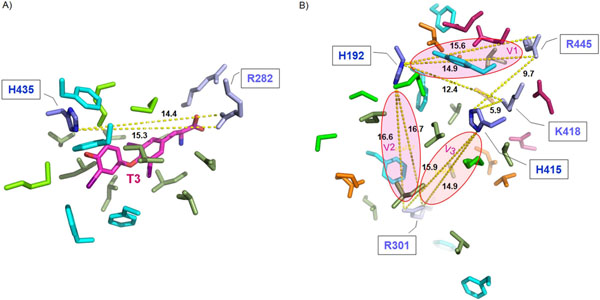
**Applications for potential modes of T3 binding at MCT8 by comparison between the crystallized hormone/receptor complex and the structural MCT8 model. A)** T3 is bound to its receptor in a specific mode characterized by several molecular interactions like H-bonds to a positively charged arginine and a histidine (figure 6A). The amino acid side chains are in a certain distance to each other in a range between 14.4 and 15.3 Å. **B)** Measurement of the distances between the histidines and positively charges residues in the MCT8 model (“inside-open” conformation) reveals three combinations of Arg-His pairings in a similar distance to each other (red translucent regions V1-V3 (V–variant)). Although the distance between R445 (highly conserved among the MCTs) and H192 is around 15 Å, some hydrophilic amino acids are in close spatial distance (V1) which would cause sterical clashes with the T3 substrate. In another transporter conformation this might be different. Of note, R445 and H192 are functionally important as demonstrated by pathogenic mutations (figure 3A). The importance of R445 for transport was also shown by directed mutagenesis.

Structure images were produced using the PyMOL Molecular Graphics System, Version 1.3, Schrödinger, LLC.

### Amino acid sequence alignment of human MCTs and the unification of residue numbering

Amino acid sequences of human MCT family members were aligned to analyze shared and divergent features in amino acid composition (figure 4). Details of the alignment procedure are recently described by Kinne and Kleinau et al. [7]. The alignment was used to highlight positions of pathogenic *MCT8* mutations. This alignment enables comparison and analyses of residue conservation among human MCTs (figure 1).

We suggested previously a unifying numbering system for MCTs similar to family A GPCRs [7]. This position identifier scheme uses a highly conserved residue in each TMH as a common reference for all members of the MCT family. The first number is related to the particular helix, the second number after the dot determines the specific position in relation to the conserved residue. For example, the highly conserved tryptophan in TMH1 is defined as 1.50 and the highly conserved proline from TMH2 is defined as 2.50 (figure 4).

## Results and discussion

### The structural MCT8 model: molecular insights and predictivity

Structural models represent potential conformations of the target macro-molecule. Because of the well known causal dependency between specific biological functions (like substrate binding or transport mechanisms at MCTs) and particular structural features, structural models are useful to guide experimental approaches or to support the interpretation of experimental data. In our recent study, we identified two functionally important amino acids located at TMH8 and TMH10 [7]. In this study we started with the hypothesis that two specific charged amino acids in the transmembrane region (R445^8.50^ and D498^10.49^) may interact with the amino acid backbone of iodothyronines. To prove this hypothesis, we have mutated both residues to alanine and tested transport of the natural substrate T3, as well as of two substrates lacking either the carboxyl or the amino group, 3,3’,5-triiodo-thyronamine and 3,3’,5-triiodo-thyroacetic acid. Both alanine mutants were inactive towards all three substrates. These findings support our assumption that both amino acids are important for substrate transport. The MCT8 model also predicted a potential salt bridge between both amino acids and an H-bond from R445 to the helix-backbone adjusting the relative orientation between TMH8 and TMH10. In conclusion, these two residues may have a dual function for substrate interaction and for the conformational switch mechanism. Interestingly, positively charged residues corresponding to MCT8 position 445^8.50^ can be found in all MCTs. This position and spatial region may bear a fundamental role, since also in other twelve helix-transporters positively charged residues are suggested or evidenced to participate in the transport mechanisms, e.g. for GlpT [8], MCT1 [13], and OATP1C1 [14]. Finally, our findings were supported by the recent report of a naturally occurring mutation at MCT8 position 445 (R445C, reviewed in [4]).

To further evaluate the reliability of our MCT8 model, we retrospectively examined other recently published functional data. An alanine scanning study focussing on all 10 cysteines within hMCT8 [16] was performed to test the contribution to homo-oligomeric MCT8 arrangements. The alanine mutants did not alter MCT8–mediated substrate transport. We have mapped these cysteines on our MCT8 model and found that none of the cysteine side chains participates directly in the substrate channel, except C283 close to the cytosolic entry side (figure 5). Accordingly, the cysteine to alanine substitutions (shorter side chains than of cysteines) did not alter properties of the transport channel and are likely not involved in transport. Finally, seven (C184, C231, C244, C281, C283, C491, C497) out of the ten cysteines are located inside the transmembrane part. Therefore, only the extracellular 3 cysteines (C436, C481, C546) in a non-reducing environment could in principle participate in intermolecular dimer-contacts via disulfide bridges. Of note, they are not in spatial distances to each other (more than 20 Å) to form intramolecular disulfide bridges.

### Naturally occurring side chain substitutions are indicators for functionally and structurally important MCT regions

We here explored the spatial localization of MCT8 mutations in the three-dimensional “inside-open” MCT8 homology model. First of all, we found by comparison of hMCT amino acid sequences and the MCT8 model that:

1. All single side chain missense mutations are located within TMHs or at helix-loop transitions (figure 2A), but not at extra- or intracellular loops. This finding highlights the transmembrane helices as most important for MCT8 function.

2. Pathogenic MCT8 mutants are localized to a high degree at conserved wild-type amino acids among MCTs (figure 4).

Conserved residues of related proteins (like the MCT family) indicate amino acids of functional and/or structural importance. Wild-type positions of MCT8 mutations at helices 5 to 12 are conserved more than 50% among hMCTs. Three out of nineteen positions are conserved to 100%, four positions are conserved to 90%. In conclusion, it can be speculated that mutations at highly conserved amino acids likely cause pathogenic dysfunction. These positions appear as essential for hMCTs and structural/functional similarities especially at these particular positions are preserved.

Furthermore, we have subdivided pathogenic mutations into two main groups (figure 1): I. mutations either at or to proline/glycine which leads likely to structural changes between helices or directly at helices (prolines in helices supporting kinks); II. side chain substitutions located close to the putative substrate channel which might directly modify substrate transport.

### Mutations at or to prolines and glycines, respectively, cause structural misfolding

Interestingly, for MCT8 the majority (7 out of 9) of proline/glycine mutations (P/G-X, e.g. P537L) or mutants to proline (X-P, e.g. L512P) are located at helices 9-12 (figure 1, figure 3A). They involve conserved amino acids among the MCTs. Prolines and glycines are known to support kinks and bulges in helices [17-19]. This has strong impact on both the helix (local) and the protein (global) structure. For that reason structural alterations of the helices by mutations at or to proline/glycine, respectively, can be assumed to cause MCT8 misfolding by modification of helix conformation. In consequence deficiencies in cell surface expression of such mutants have been observed [10]. Additionally, for the P/G-X mutants it can be assumed that substitutions of prolines or glycines with enlarged side chains like leucine are predestined to cause sterical clashes in a tight micro-environment of interacting side chains.

### Pathogenic MCT8 mutations indicate important determinants and regions for substrate transport

Wild-type amino acids of those mutants that are close to the transport channel (figures 2A and 3B) might be directly or indirectly involved in substrate transport mechanisms. Interestingly, amino acids H192, S194, G221, A224, and R445 are located at a similar spatial layer which lies at the closed bottom substrate channel viewed from the intracellular side (figures 3B and 5B). These residues are in a specific region that must be modified or “opened” for substrate transport. Therefore, they might be involved in the switching mechanism between an “inside-open” and “outside-open” conformation. Particular residues here may function thereby as gate keepers as already described for R445 [7]. Interactions between neighbouring helices as suggested for R445 may constrain the “bottle neck like” conformation of this region. During the conformational switching such constraints must be re-arranged to enable structural movements of the helices. However, it needs more experimental characterization to clarify the detailed molecular involvement of each of these wild-type amino acids, but we can assume that the pathogenic substitutions do not maintain MCT8 transport function.

### Potential T3 binding sites in the MCT8 substrate transport channel

One may assume that MCT8 substrates bind along the open channel-part in the “inside-open” MCT8 model. In order to identify amino acids which may contribute to substrate binding and transport, we used a comparative strategy. The previously published crystal structure of the T3-bound thyroid hormone receptor (TR) beta ligand binding domain (PDB code 3GWS [20]) reveals detailed insights in T3 binding (figure 6A). Surrounded by hydrophobic aliphatic and aromatic amino acids, T3 is bound by H-bonds to an arginine and a histidine (figure 6A). Side chains of these two residues are in a defined spatial distance to each other of around 14-15 Å (figure 7A). Assuming a certain degree of analogy in T3 binding, such specific residues, their biochemical properties and their arrangement in the binding pocket, might represent a typical pattern for T3 binding. In the MCT8 homology model two histidines (H192^1.67^, H415^7.53^) and three positively charged residues (R445^8.50^, K418^8.56^, R301^5.39^) are predicted to point towards the substrate transport channel (figure 6B). Measurement of the side chain distances between the histidines and positively charged residues (“inside-open” MCT8 conformation, figure 7B) reveals three combinations of Arg-His pairs at a similar distance to each other as in the T3-TR beta complex. These pairs and distances are shown in figure 7B as potential binding regions for T3 in the channel cavity. Of note, although the distance between R445 (highly conserved among the MCTs) and H192 is around 15 Å like in the T3 receptor, few other amino acids are in close spatial proximity likely would lead to sterical clashes with substrate in this conformation. In an “outside-open” conformation this might be different.

Nevertheless, R445 and H192 have already been shown to be functionally important (references in [7]). The present comparative analysis suggests two new potential key-players for substrate transport: R301^5.39^ (highly conserved among the hMCTs 1-13) and H415^7.53^ (conserved in hMCT8, hMCT10, hMCT11, and hMCT13). Both amino acids have not yet been investigated experimentally. R301 at TMH5 is, in contrast to the two other positively charged residues inside the channel, localized more closely to the intracellular side. Noticeably, in all three potential variants of T3 binding modes always one amino acid is linked to the potential “switching sensitive” region at the closed bottle neck of the transport channel.

## Conclusions

Naturally occurring mutations in MCT8 provide molecular insights into protein regions important for protein folding and the switching mechanism during substrate transport. This was shown here by the assignment of mutations especially at TMH9 to TMH12 leading to MCT8 misfolding. Furthermore, by highlighting specific positions of pathogenic mutations in the three-dimensional structure, we have specified a critical region for the transition between the postulated “inside-open” and “outside-open” conformations. These amino acids are located at a bottle neck of the transport channel cavity, surrounded by substrate binding-sensitive amino acids. Our MCT8 model has guided several new insights in the relationship between sequence, structure and function of this transporter. This model is in accordance with experimental data and is predictive to support the identification of functional key-player amino acids. Future studies based on this model are aimed to clarify unknown details of molecular events at MCT8 and likely at other members of the MCT family. This includes: 1. identification of the complete set of transport-sensitive residues, 2. description of structural re-arrangements, 3. identification of binding-sensitive residues, 4. details of ligand binding with molecular explanation of substrate specificity. Finally, for comprehensive understanding the relevance of oligomeric MCT8 constellations must be considered.

## Competing interests

There are no competing interests.

## Authors’ contributions

GK wrote the draft, performed and designed the studies, analyzed the data, prepared the figures; US analyzed the data, wrote the draft; AK analyzed the data; JK performed and designed the studies; AG data evaluation and discussion; HK analyzed the data, project coordination; HB analyzed the data, project coordination. All authors drafted the manuscript and approved the final manuscript.
